# Managing the COVID-19 risk: the practicalities of delivering high stakes OSCEs during a pandemic

**DOI:** 10.15694/mep.2020.000173.1

**Published:** 2020-08-19

**Authors:** Claire Ann Canning, Kirsty Jane Freeman, Ian Curran, Katharine Boursicot

**Affiliations:** 1 Duke-NUS Medical School

**Keywords:** OSCE, Clinical Examination, Pandemic, COVID-19, Simulation, Clinical Skills Assessment, Performance Skills Assessment, Quality Assurance, Assessment

## Abstract

This article was migrated. The article was marked as recommended.

The impact of the COVID-19 pandemic on the teaching and assessment of clinical skills continues to pose significant challenges for healthcare education providers worldwide. In March 2020 Duke-National University of Singapore (Duke-NUS) Medical School demonstrated how to design and implement clinical skills examinations (OSCEs) in the early phase of COVID-19. As governing bodies continue to revise restrictions to help ‘flatten the curve’, educational institutions have to undertake a rapid review of assessment practices and adapt to this ever-changing environment. This case study describes the risk-assessments and challenges faced when delivering high stakes OSCEs during the COVID-19 pandemic. We also describe successful mitigation strategies implemented to combat these risks, and how we also embraced and leveraged technology in a very creative way despite the restrictions and constrained environment.

We describe and share practical guidance that may be of help or interest to healthcare education providers across all disciplines on how to effectively deliver clinical and procedural skills examinations that are authentic, valid and comply with the strict national COVID-19 restrictions implemented during this time.

## Background

Duke-NUS Medical School is a US-style graduate-entry medical school of the National University of Singapore offering a Doctor of Medicine (MD) programme. As per the assessment strategy at Duke-NUS Medical School any final year medical student who is unable to achieve a passing grade in the final OSCE is required to undertake a supplementary examination to demonstrate competence and progress to graduation. For the class of 2020 the main OSCEs were held in March. Boursicot and colleagues describe how these final level OSCEs were conducted in their paper “Conducting a high-stakes OSCE in a COVID-19 environment” ( Boursicot et al., 2020). However, with the evolving pandemic and further control measures or restrictions due to COVID-19, the practices applied in March 2020 no longer reflected the risk mitigation strategies we were required to adopt eight weeks later. This case study describes the enhancements, considerations and approaches we put in place when running the supplementary high stakes OSCEs. Specifically, we discuss the mitigation strategies implemented to combat these risks, and how we embraced and leveraged technology in a very constrained environment.

Since the outset of the COVID-19 outbreak, the Singapore government has implemented a comprehensive range of necessary, national control measures to reduce community spread of the SARS-coronavirus 2. These control measures are mandatory, legally enforced and have evolved over time to reflect emerging risk and understanding of the SARS-coronavirus 2. Key principles include a focus on strict infection control and personal protection along with an ‘elevated set of safe distancing measures’. The most rigorous, referred to as a ‘Circuit Breaker’ from 7 April 2020 until 4 May 2020 ( Ministry of Health, 2020) amounting to a national lockdown. On 21 ^st^ April 2020 the government announced that the Circuit Breaker (CB) would be extended by another four weeks until 1 ^st^ June 2020 (inclusive). CB measures required the population to stay at home, avoid non-essential travel and limit face to face interactions. The supplementary OSCEs were scheduled to occur on 5 ^th^ May 2020 so we had to rapidly review and enhance our planning to address these further challenges and restrictions.

The final year OSCEs are essential to allow students to graduate and commence their post graduate training (PGY1) in June 2020. Faculty buy-in, support from regulatory bodies and utilizing available resources are essential for the successful conduct of high stakes assessments ( Sabzwari, 2020). This paper provides practical guidance on how to deliver clinical and procedural skills examinations (in an OSCE format) that are authentic, valid, and comply with the strict national COVID-19 restrictions implemented during these unprecedented times.

## Risk mitigation

The safety of all involved in this OSCE was our number one priority. The School engaged with the Medical School Safety Team, the National University of Singapore Provost, the Ministry of Health (MOH) and Ministry of Education (MOE), to identify, assess and mitigate risk when planning and running these high stakes OSCEs. We were required to seek formal approval for our plans from the University and the Ministries of Health and Education. All stakeholders supported the running of these examinations to give our students an attempt to graduate and crucially join the frontline healthcare workforce.

As part of the COVID-19 control measures the following risk mitigation strategies were implemented:

• All attendees were required to wear a mask during commuting, and whilst on campus
• All attendees were encouraged to travel using point to point transport service (e.g. taxi or private vehicle)
• Staggered arrival time of attendees to ensure group cohorting, smooth arrival at the front entrance, and avoidance of congregation
• All examiners were recruited from a single institution
• Temperature was checked on arrival
• Travel & Health Declarations completed by all parties coming to campus
• Movement of attendees confined to the examination venue only
• Hand sanitiser stations located at simulation centre entrance, outside and inside each clinical examination room
• Use the Trace Together APP, a Singapore government smart phone app designed to use Bluetooth tracking to facilitate and support contact tracing of individuals if required
• Practice safe social distancing and strict personal hygiene measures
• Disinfection of surfaces and equipment in-between each student

## Manpower

Key to the success in delivering a high stakes OSCE in a pandemic is to empower/engage an agile nimble team. Facilitating high stakes OSCEs is notoriously labour intensive and gregarious activity, personnel on-site can include examiners, real and/or simulated patients (SPs), assessment faculty and support staff. In view of the national restrictions, the decision was made to eliminate face to face interactions wherever possible, by supporting the clinical examiners to assess students from off-site locations, thus enabling an overall reduction in the total number of individuals physically on-site.

Within a 10-day window the assessment and clinical performance teams overhauled and revised a robust operational strategy to safely and effectively run high stakes OSCEs, adhering to five key principles:

1. Minimise in person student:examiner interactions
2. Minimise the number of SPs on-site
3. Minimise the number of faculty/staff on-site
4. Test the technological solutions robustly
5. Familiarise all participants with the new arrangements

The OSCE included stations where students had to take a clinical history and summarise their findings, physically examine a patient, and a range of professional encounters such as explaining diagnoses/management to patients and demonstrate practical skills using manikins with SPs. The latter hybrid stations comprised the SP who provided the professional context of patient interaction as well as the manikin on which the practical skill was performed.

It was deemed that 10 out of a total of 12 stations were conducive to having examiners score offsite, decreasing the total number of on-site examiners from 12 to 2. Four of the stations were conducive to simulated patients participating remotely (from their homes), such as clinical history taking and explanation stations, which resulted in reducing the total number of SPs onsite from 12 to 8. It was decided that the two physical examination stations required the student to be present on-site to perform the clinical examination, with the clinical examiner in the room. To decrease the number of staff on-site, we reviewed the tasks (not individuals) required to successfully, and safely and effectively run these examinations. With the task list identified, each task was then categorized as on-site or off-site. It was decided that 4 staff would be required to facilitate the on-site tasks, with 6 staff supporting off-site.

With minimal staff on-site to undertake the numerous tasks required to operate the practicalities of the OSCE, there was a requirement to upskill staff in several key areas. For example, staff from the assessment team were trained to swap over the clinical skills stations consumables and reset the manikins in the break between students. All members of the team were required to upskill on the Zoom video-conferencing platform to be able to troubleshoot any issues that may arise during the examination.

## Embracing technology

Planning for OSCEs usually occurs over many months, however the unprecedented arrival of COVID-19 required the team to be dynamic and agile as the level of restrictions constantly changed often at short notice. Alexander and colleagues stated the “The pandemic should not be a time of educational stasis, it is a chance to harness the power of technology and novel educational tools”, and the same should be said for assessment ( Alexander et al., 2020). Ten days prior to the examination our challenge was to embrace technology and adapt rapidly to the new requirements whilst maintaining the authenticity and validity of these high stakes supplementary OSCEs.

The first practical challenge was to enable observation of students performing clinical procedural skills stations via video conferencing software, providing the maximal level of detail to enable an off-site examiner to successfully score the interaction. The video conferencing platform adopted for this examination was Zoom Video Communications Inc. We chose Zoom because staff and students were familiar with it due to recent e-learning practices.

**Figure 1.**
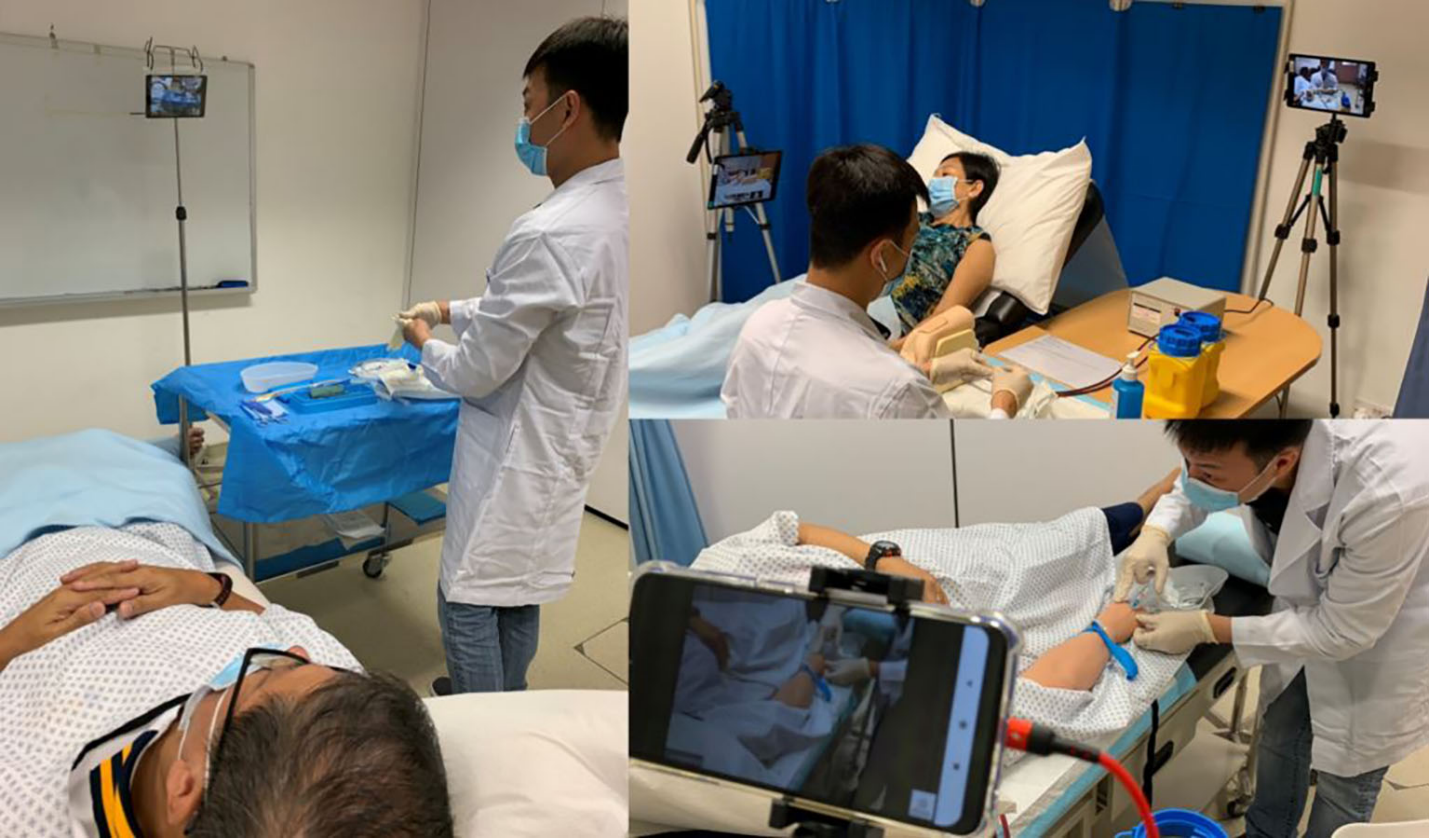
Configuration of Simulated Patient, candidate and cameras to facilitate off-site examination.

During COVID-19 restrictions, and due to the short timelines, we were not able to procure extra audiovisual devices to enable live streaming of patient/student interaction required for examiner scoring. We adopted the ‘under the stairs approach’ making use of the resources we had at hand. Resources at hand were iPads, mobile phones, and laptops with webcam. The challenge was where and how to place devices in each room to enable them to capture a global view and a close-up view of the skill being performed. With duct tape, a selfie stick, two tripods, three IV poles, plastic sheet protectors and countless cable ties, we positioned the cameras in each room to ensure the examiner was able to view the students’ performance adequately ( Figure 1).

## Final thoughts

This paper provides practical guidance on how to deliver high stakes OSCEs during a pandemic. Whilst we highlight opportunities where stations could be rapidly converted to remote stations, so having examiners and SPs participate off-site, it is also important to note that for some stations, particularly the physical examination stations, face to face interactions were deemed essential for validity and authenticity. The clinical examiners also had to examine the patients to calibrate and confirm the clinical findings so they could fairly assess the performance and confirm the findings of the candidate.

Faculty buy-in is key to the successful implementation of innovative on-line OSCEs. Paramount in our activities was ensuring staff, students and simulated patients were safe. By engaging with all our key stakeholders, including students, clinical faculty, administrative staff and University and Ministry leaders we were able to ensure the safety of all, whilst maintaining the authenticity and validity of these high stakes examinations. Over 8 weeks after the conduct of the OSCE we have had no reported episodes of student, examiner or staff infection with COVID-19.

## Take Home Messages

• Conduct a thorough risk assessment and tailor the risk mitigation strategies to address the prevailing risks and control measures.
• Expand the scope of practice of team members, be agile in your approach and enable transfer of skills across teams
• Embracing technology does not have to be expensive - review resources at hand and use cost effective solutions such as duct tape and cable ties
• Engage all stakeholders early and often - government agencies, university executives, examiners, simulated patients and students
• Ensure adequate personal protective equipment for stations that require the students to interact with SPs

## Notes On Contributors

**Claire Ann Canning,** Assistant Professorfor Assessment and Progression, Duke-National University of Singapore Medical School, Singapore. Main author, and along with Kirsty Jane Freeman conceptualized, wrote and revised manuscript based on comments and suggestions from the other authors.

**Kirsty Jane Freeman,** Lead Associate for Simulation, Duke-National University of Singapore Medical School, Singapore. Along with Claire Ann Canning conceptualized, wrote and revised manuscript based on comments and suggestions from the other authors.

**Ian Curran**, Vice-Dean for Education, Duke-National University of Singapore Medical School, Singapore. Reviewed draft, advised on national level and institutional directives.

**Katharine Boursicot,** Associate Dean for Assessment and Progression, Duke-National University of Singapore Medical School, Singapore. Contributed to the conceptualization of the paper, reviewed and revised drafts.
