# Supplementary material for: Managing the COVID-19 risk: the practicalities of delivering high stakes OSCEs during a pandemic
Source: MedEdPublish (2016). 2020 Aug 19;9:173. [Version 1] doi: 10.15694/mep.2020.000173.1 (PMC10702641; doi:10.15694/mep.2020.000173.1)
Supplement: Peer review history

# Reviewer report: Reviewer response for version 1

**DOI:** 10.21956/mep.20132.r31074

**Published:** 2020-08-30

**Authors:** Ronald M Harden

**Affiliations:** 1 AMEE

This review has been migrated. The reviewer awarded 5 stars out of 5 I have argued that one reason for the OSCE's popularity is that the approach can be adapted to suit different needs. This useful paper demonstrates what can be achieved with imagination, creativity, attention to detail and the cooperation of the stakeholders in response to the present Covid constraints.A highly recommended read for all interested in education in the health professions.

Reviewer Expertise:

NA

No decision status is available

# Reviewer report: Reviewer response for version 1

**DOI:** 10.21956/mep.20132.r31075

**Published:** 2020-08-28

**Authors:** Richard Fuller

This review has been migrated. The reviewer awarded 5 stars out of 5 This is a welcome follow up to the initial paper by the Duke-NUS group, and provides practical (and road tested) advice to those needing to run an OSCE in a variety of lock down situations.Some really important take home messages for me1) A radical rethink about some station formats. Does the examiner need to be there in person? Can we simulate a video consultation?2) Rethinking ''Technology' - simpler solutions are more cost effective, and more palatable to stake holdersSome interesting questions for the future...!1) With examiners remote from the interaction, how does this affect candidates behaviour. Less 'performance' and more authenticity - vs the difficulty of presentation to a screen to summarise? Really interesting research possibilities2) The use of video scoring in OSCE - for readers who want to explore more, a series of paper from Keele in the UK give some interesting perspective - e.g. https://dx.doi.org/10.1080/0142159X.2020.1801997

Reviewer Expertise:

NA

No decision status is available

# Reviewer report: Reviewer response for version 1

**DOI:** 10.21956/mep.20132.r31077

**Published:** 2020-08-25

**Authors:** Sandra Kemp

**Affiliations:** 1 Curtin Medical School, Curtin University

This review has been migrated. The reviewer awarded 5 stars out of 5 This is an interesting paper. It clearly describes a way of approaching risk assessment and mitigation strategies for an OSCE. I think the practical guidance the paper provides will be useful to readers who are trying to conduct performance assessments in these Covid constrained times. I thought the description of the process for decision making around what could be tested onsite versus offsite/remote was also very helpful. I would recommend this paper to others who are involved in high-stakes OSCEs.

Reviewer Expertise:

NA

No decision status is available

# Reviewer report: Reviewer response for version 1

**DOI:** 10.21956/mep.20132.r31078

**Published:** 2020-08-21

**Authors:** Trevor Gibbs

**Affiliations:** 1 AMEE

This review has been migrated. The reviewer awarded 5 stars out of 5 A very interesting and practical paper at a time of almost "too-many " Covid papers. A slightly different approach when compared to the previous OSCE paper by Boursicot et al. I specifically appreciated the risk assessment and the tailoring the risk mitigation suggestions, something that we all need to think about these days. Certainly a paper for everyone involved in high-stakes OSCE examinations.

Reviewer Expertise:

NA

No decision status is available

# Reviewer report: Reviewer response for version 1

**DOI:** 10.21956/mep.20132.r31076

**Published:** 2020-08-20

**Authors:** Trudie Roberts

**Affiliations:** 1 Leeds Institute of Medical Education

This review has been migrated. The reviewer awarded 5 stars out of 5 Although like many others I'm feeling a little 'all CoVided out' by the number of papers purporting to give a new perspective on "Education in a Time of CoVid". I do think that practical guidance drawn from actual experience is very useful. This is what the authors of this paper provide. So I recommend this paper to others who are running or trying to run performance based assessments in the current climate are a clearly written and practical submission.

Reviewer Expertise:

NA

No decision status is available
